# Numerical Investigation of a Microfluidic Biosensor Based on I-Shaped Micropillar Array Electrodes

**DOI:** 10.3390/s24248049

**Published:** 2024-12-17

**Authors:** Maliheh Azimi Roueini, Amal Kabalan

**Affiliations:** Department of Electrical and Computer Engineering, Bucknell University, Lewisburg, PA 17837, USA; mar059@bucknell.edu

**Keywords:** microfluidic biosensor, COMSOL Multiphysics, micropillar array

## Abstract

Micropillar array electrodes offer several advantages, such as enhanced mass transport, lower detection limits, and the potential for miniaturization, making them instrumental in the design and fabrication of electrochemical biosensors. The performance of these biosensors is influenced by electrode geometry, including parameters like shape and height, which affect surface area and overall sensitivity. In this study, we designed a microfluidic electrochemical biosensor featuring micropillar array electrodes, modeled in COMSOL Multiphysics. We compared the response currents of I-shaped and cylindrical micropillar array electrodes. The working electrode (WE), consisting of 100 micropillars with a height of 300 µm, exhibited a sensitivity of 1.61 µA·cm^2^·mM^−1^ in cyclic voltammetry, highlighting its effectiveness for analyte detection.

## 1. Introduction

*Moraxella catarrhalis* is a Gram-negative diplococcus and a significant respiratory tract pathogen. It is one of the leading causes of otitis media in children and the second most common cause of exacerbations in adults with chronic obstructive pulmonary disease (COPD) [1,2]. This bacterium belongs to the family Moraxellaceae and can cause infections in the respiratory system, middle ear (otitis media), eyes, central nervous system, and joints [3]. Conventional methods for identifying *Moraxella catarrhalis* include culturing, Gram staining, and biochemical tests such as PCR and enzyme-linked immunosorbent assays (ELISAs). While these methods are sensitive and robust, they have disadvantages, including contamination and false-positive results. Additionally, they often require a large number of samples and extensive preparation, which increases time-to-results (TTR) and costs [3,4,5]. *M. catarrhalis* is also difficult to obtain from children since obtaining specimens from the inner ear, nose, throat, and lungs may require invasive or painful methods. The inner ear is particularly difficult to obtain a specimen from. It is because of this that most ear infections are treated empirically for pathogens that are expected to be causing the infection. The nose and throat are more accessible as far as obtaining samples is concerned, but the lungs (lower respiratory tract) are particularly difficult to access as well. Sputum samples often can be used if sputum is produced, but children who are infected with *M. catarrhalis* are usually of such an age that they cannot easily cough sputum for a sample. Because of this, the only alternative to sputum samples that could yield a definitive organism (*M. catarrhalis*) in children with respiratory infections would be bronchoscopy.

Bronchoscopy is a procedure whereby a flexible scope is inserted into the airway of the patient and used to inspect the lungs/lower airways and obtain mucus samples. Because this is an invasive procedure, children need to be sedated in order for this procedure to be performed safely. Therefore, in most situations, lower respiratory infections, such as bronchitis and pneumonia, are treated empirically for the pathogen that most commonly cause the infections (*M. catarrhalis* and others). In some situations when bacteria are resistant to empiric antibiotics or if the patient is not responding to empiric antibiotics, bronchoscopy is used. In other situations, such as with protracted bacterial bronchitis, *M. catarrhalis* causes an indolent infection that has not been treated with antibiotics or treated adequately. In this situation, bronchoscopy is needed to diagnose this condition so that the right course of antibiotics can be prescribed. A less invasive means of making such a diagnosis would be of great benefit to the patient and reduce the cost of medicine.

The proposed approach tends to provide a quicker way of diagnosing this pathogen and providing treatment accordingly. To our knowledge, there is no device available that can detect *M. catarrhalis* using a biosensing mechanism. Thus, this interdisciplinary work between engineering and the medical field will enhance the well-being of patients by rapidly diagnosing *M. catarrhalis* and providing treatment to prevent its spread.

One promising method is electrochemical detection, which has gained increasing interest [5,6]. Electrochemical biosensors convert chemical signals into electrical signals and consist of an analyte, receptors, a signal transducer, and a data analysis system [7]. The electrode, which is functionalized to capture specific antibody–antigen interactions, generates a digital signal in the form of potential or current. This component is crucial for the electrochemical biosensor platform [8]. Advances in electrode design and fabrication have enhanced sensitivity and reduced detection time [7]. For instance, nano-structuring and microarrays increase the reaction area, improving biosensor performance [9]. Compared to conventional electrodes, micropillar array electrodes offer several advantages, including high-quality signal transmission, low ohmic loss, improved limit of detection, and higher response current. These benefits contribute to their growing popularity [10,11]. Additionally, micropillar array electrodes enhance interactions between target molecules and the surface, reducing both the limit of detection and the detection time [9,11]. Integrating electrodes into microfluidic systems further improves the performance of electrochemical biosensors by decreasing analysis time, requiring smaller sample volumes, and increasing sensitivity [12].

Currently, various scientific efforts are underway to develop micropillar array electrodes to enhance the sensitivity, limit of detection, and overall performance of electrochemical biosensors. In 2009, Ginoza et al. introduced an electrochemical biosensor featuring micropillar structures on the working electrode, which significantly improved collection efficiency and detection sensitivity, resulting in a 22-fold increase in current response compared to flat electrodes [13]. In 2019, Hu et al. developed an electrochemical sensor with micropillar array electrodes, demonstrating that these structures increased surface area and led to satisfactory analytical performance for detecting lead (Pb) [10]. In 2020, Chen et al. studied a biosensor consisting of 90 micropillars both numerically and experimentally, finding that it exhibited 1.5 times greater sensitivity than a planar electrode [14]. Liu et al. also introduced a microfluidic biosensor in the same year with a variable number of pillars on the working electrode, showing a significantly higher current response under flow conditions compared to planar microelectrodes, regardless of the micropillar shape [11]. Additionally, Ali et al. developed a microfluidic biosensor in 2021 with a 10 × 10 micropillar configuration to detect COVID-19 within seconds, employing gold micropillar array electrodes to enhance sensitivity and reduce detection time. They reported limits of detection for antibodies to the SARS-CoV-2 spike S1 protein and its receptor-binding domain (RBD) at 2.8 × 10^−15^ m and 16.9 × 10^−15^ m, respectively [15].

This work presents a novel microfluidic biosensor featuring I-shaped micropillar array electrodes designed to improve the detection of *Moraxella catarrhalis* as a respiratory pathogen. Cyclic voltammetry was employed to investigate the effects of flow rate and calculate sensitivity. Our findings demonstrate that using I-shaped micropillar array electrodes significantly increases current density and enhances the operational performance of the biosensor.

## 2. Materials and Methods

In microarray electrodes, the velocity vector and concentration gradient facilitate convection and diffusion, respectively, which together describe the mass transfer of electroactive species in a microchannel [11,16] as shown in (1):

(1) $$\frac{\partial C}{\partial t}=D\nabla^{2}C-u.\nabla C$$

Here, *C* and *D* represent the concentration of the analyte and the diffusion coefficient, respectively, while *u* denotes the flow velocity. We assume a fully reversible electrochemical system for this analysis [17]:

O + e^−^ ⇆ R (2)

where *O* is the oxidized species, and *R* is the reduced species. The Butler–Volmer equation is utilized to calculate the current density for this redox reaction:

(3) $$i=nFA(k_{f}C_{o}\left(t\right)-k_{b}C_{R}\left(t\right))$$

*F* is Faraday’s constant; *A* is the area of the electrode; *C_O_(t)* and *C_R_(t)* are the concentration of the analyte at the electrode surface at time *t*. In addition, *k_f_* and *k_b_* are the forward and reverse reaction rate constants, which can be expressed as

(4) $$k_{f}=k_{o}exp(-\alpha \frac{F}{RT}\left(E-E^{0\prime }\right))$$

(5) $$k_{b}=k_{0}exp((1-\alpha )\frac{F}{RT}\left(E-E^{0\prime }\right))$$

*k*_0_, *α*, *R*, *T*, *E*, *E*^0^ show the standard heterogeneous rate constant, the transfer coefficient, the gas constant, the absolute temperature, the potential applied to the electrode, and the equilibrium potential, respectively [11,17].

## 3. Model Definition

Using micropillar array electrodes enhances electrical signal output due to their significantly larger surface area compared to planar electrodes of the same projected area [14]. The dimensions of the micropillars—such as diameter, height, and spacing—considerably influence their overall surface area [11]. In this study, we investigate how variations in the shape, number, and dimensions of the micropillars affect the design of the working electrode (WE). To achieve this, we conducted a 3D simulation using the electroanalysis module of COMSOL Multiphysics software. In this module, the current density for the reaction is described by the Butler–Volmer equation, which accounts for the electroanalytical processes involved. The total current recorded can be obtained by integrating the local current density across the electrode surface. This electroanalysis interface defines an electrode current that varies according to

(6) $$I_{e1}=\int i_{1OC}dA$$

**Figure 1:**
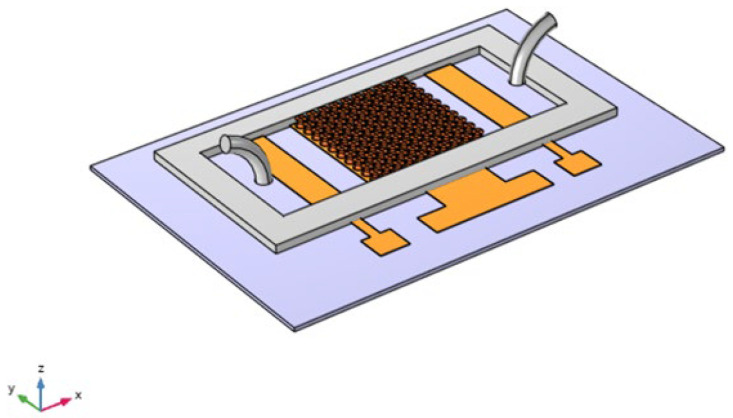
Schematic of the sensing device (RE: reference electrode, WE: working electrode, CE: counter electrode).

Figure 1 illustrates the design of the biosensor, which comprises a working electrode with micropillars, an Ag/AgCl reference electrode, and a platinum auxiliary electrode. The biosensor features curved pillar surfaces that are functionalized with antibodies specifically designed to selectively capture analytes from the liquid sample. This design enhances the biosensor’s efficiency by maximizing surface area for binding and improving the kinetics of interaction. The bound biomolecules generate an electrical signal that varies with their local concentration at the micropillar surfaces [18].

**Figure 2:**
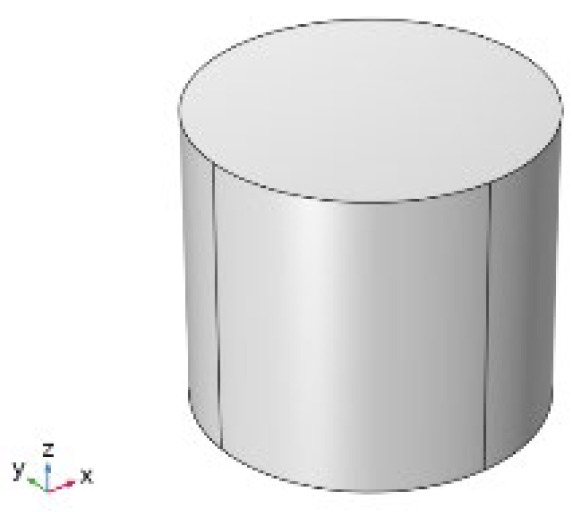
Cylindrical pillar.

At first, cylindrical pillars were considered on the WE to study the effects of the micropillars’ shape, as shown in Figure 1. We designed the WE with several configurations, from 4 × 4 to 10 × 10, and compared the changes in the response current, as shown in Figure 2.

**Figure 3:**
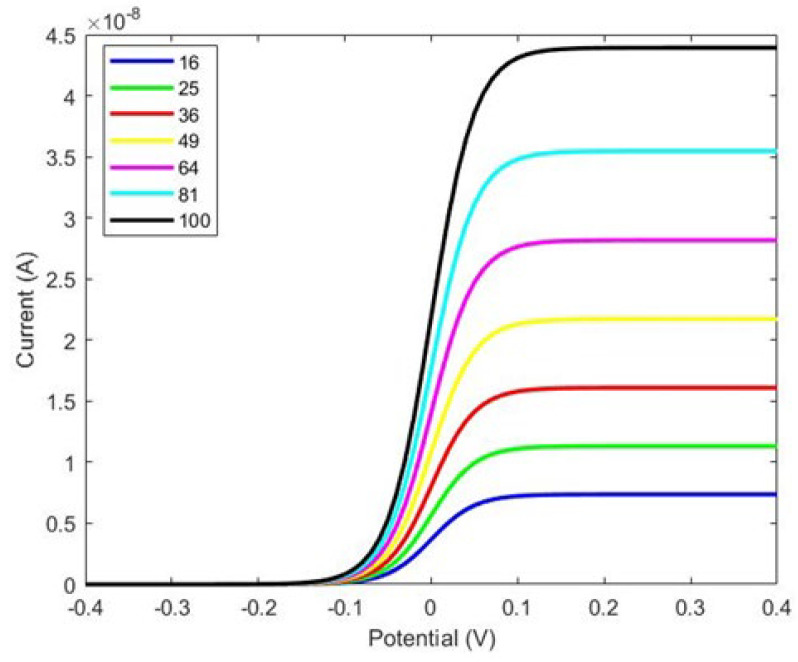
The total current as a function of applied voltage (V) from various array designs, ranging from 4 × 4 to 10 × 10 cylindrical pillars.

Figure 3 illustrates that the current is directly proportional to the surface area of the working electrode. Specifically, increasing the number of micropillars enhances the current density due to the larger area available for charge transfer. However, while the current density increases with the number of pillars, the overall current range displayed in Figure 2 is not ideal. Notably, increasing the number of pillars and the size of the working electrode does not significantly affect the total current. To address this limitation and enhance the response current detected by the biosensor, we designed a working electrode with I-shaped pillars, as shown in Figure 3. This design aims to optimize the sensor’s performance by potentially increasing the interaction area and improving binding efficiency.

**Figure 4:**
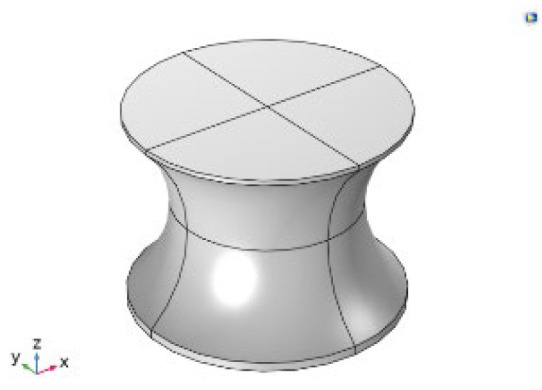
I-shaped pillar.

**Figure 5:**
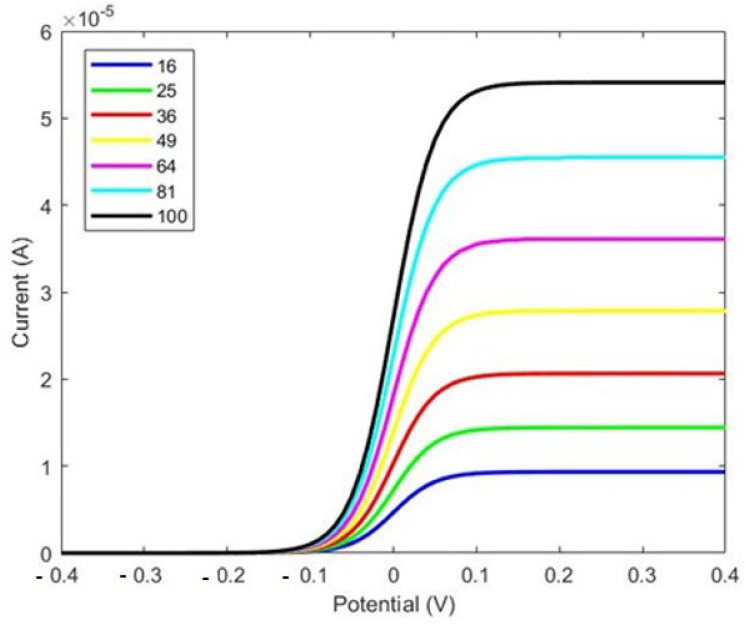
Plots of total current obtained from different array configurations varying from 4 × 4 to 10 × 10 I-shaped pillars as a function of applied voltage (V).

As shown in Figure 5, there is a considerable improvement in the response current with the electrode featuring I-shaped micropillars shown in Figure 4. A comparison between Figure 3 and Figure 5 reveals that the response current in the electrode with I-shaped micropillars is nearly 1000 times larger than that of the cylindrical micropillar electrode. In the cylindrical pillar, the current is 4.4 × 10^−8^ A for 16 × 16 pillars compared to 6.5 × 10^−5^ A in the I-shaped pillars. This increase highlights the enhanced performance of the I-shaped design, likely due to factors such as increased surface area and improved biomolecule binding efficiency.

**Figure 6:**
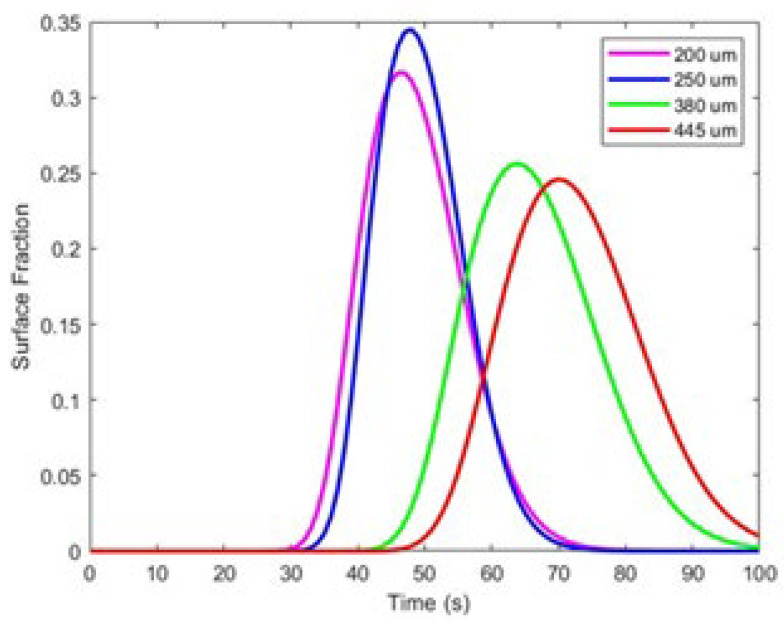
Adsorption on the surface of the (10 × 10) pillars with different spacing.

After designing the shape of the pillars, the spacing between them emerged as a critical factor for optimizing adsorption efficiency. Numerical simulations were conducted to investigate the effects of pillar spacing on adsorption levels. In this study, the spacing was varied between 200 and 450 µm. The results presented in Figure 6 indicate that optimal adsorption occurs at 250 µm. Furthermore, as the spacing between the pillars is increased from 250 µm to 380 µm, the percentage of adsorption decreases significantly. Therefore, we consider 250 µm to be the optimal spacing for enhancing the performance of the surface electrode in our biosensor design.

## 4. Results

**Figure 7:**
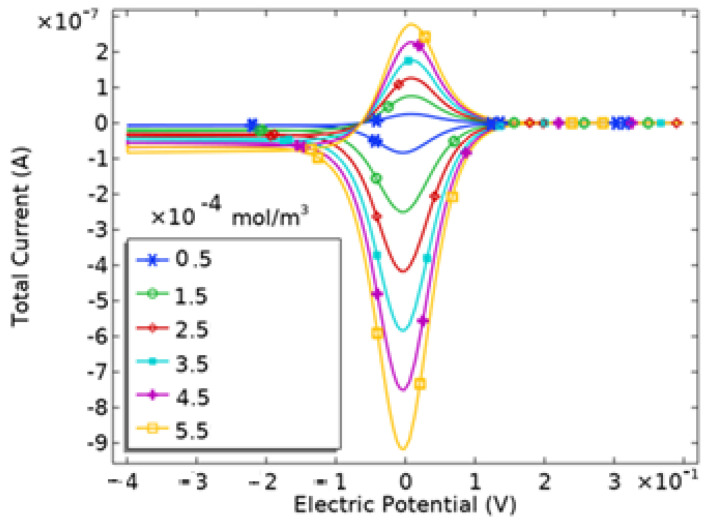
Cyclic voltammetry as a function of different concentrations.

Cyclic voltammetry (CV) was conducted to evaluate the electrochemical characteristics of the structure, recording the current response as the potential varies. We employed the electrochemistry module in COMSOL Multiphysics, which applies the Randles–Sevcik equation (Equation (7)). This equation is a key electrochemical model used to describe the relationship of peak current i_p_ (A) with other parameters [10,19].

(7) $$i_{p}=2.69\times 10^{5}.n^{3/2}.v^{1/2}.D^{1/2}.A.C$$

where *n* represents the number of electrons transferred in the redox reaction, *A* is the electrode surface area in cm^2^, *D* denotes the diffusion coefficient of the oxidized analyte in cm^2^/s, *C* is the analyte concentration in mol/cm^3^, and ν is the scan rate in V/s. The cyclic voltammetry (CV) scan was conducted within a potential range of −0.4 V to +0.4 V. As illustrated in Figure 7, we show the variation in peak currents across different analyte concentrations. With a constant scan rate and electrode area for all CV measurements, changes in analyte concentration result in corresponding shifts in current peaks. The data reveal a linear increase in current with rising analyte concentration.

**Figure 8:**
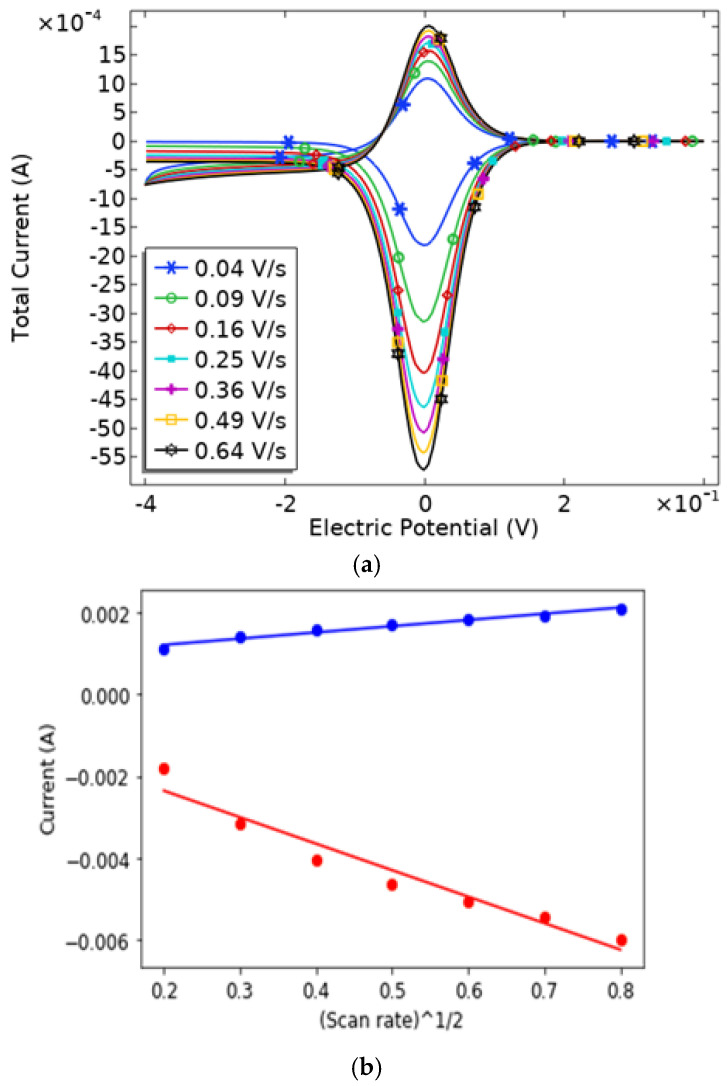
(**a**) Cyclic voltammetry applied on an electrode with microarrays with scan rates shown in (**b**).

We further explored the relationship between scan rates and redox peak currents to analyze the behavior of the micropillar arrays, as shown in Figure 8. As shown, both cathodic and anodic peak currents vary linearly with the square root of the scan rate, in alignment with the Randles–Sevcik equation, which predicts a direct proportionality between peak current and scan rate. This linear relationship indicates a diffusion-controlled kinetic process for the microarray electrodes, characteristic of a reversible electrochemical reaction [14,20]. Our findings demonstrate strong linearity for both cathodic (adjusted R^2^ = 0.9953) and anodic peak currents (adjusted R^2^ = 0.9959R), with current increasing as scan rate rises.

**Figure 9:**
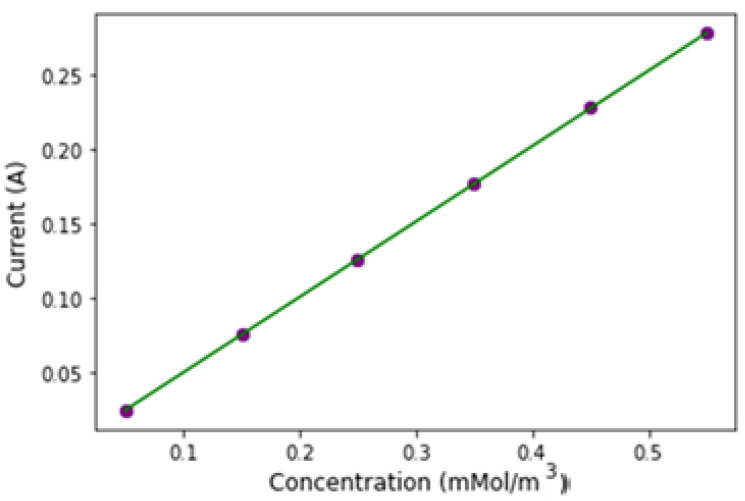
Calibration curve of the peak current vs. concentration.

The data in Figure 7 were used to determine the biosensor’s sensitivity. Sensitivity for an electrochemical biosensor is calculated by dividing the slope of the calibration curve by the electrode’s projected area [14,21]. The peak current of the microarray electrode showed a linear relationship with analyte concentration, described by the linear regression equation y = a + bx, where a = 6.76 × 10^−5^ and b = 0.506. Here, y represents the peak current (Ip, μA), and x represents analyte concentration (μM), with a strong correlation (R^2^ = 0.99) as shown in Figure 9. Based on the calibration curve, the biosensor’s sensitivity is 1.61 μA·cm^−2^ [22].

## 5. Conclusions

This study presents a numerical model developed in COMSOL Multiphysics to design a novel biosensor featuring I-shaped pillar array electrodes for detecting *Moraxella catarrhalis*. Given the need to detect even the lowest pathogen concentrations in patients, designing a highly sensitive biosensor is crucial. Here, we examined how the shape of electrode pillars influences the response current, demonstrating that I-shaped pillar array electrodes provide significantly higher response currents than cylindrical ones. Specifically, the output current of I-shaped pillars was approximately 1000 times greater than that of cylindrical pillars. Additionally, cyclic voltammetry was used to analyze the biosensor’s behavior and measure its sensitivity, which was found to be 1.61 μA·cm^−2^mM^−1^. Moving forward, the next phase involves fabricating the designed structure using 3D printing and conducting experimental validation.

## Data Availability

The original contributions presented in this study are included in the article. Further inquiries can be directed to the corresponding author(s).
